# Computed Tomography Colonography Technique: The Role of Intracolonic Gas Volume

**DOI:** 10.1155/2013/517246

**Published:** 2013-12-18

**Authors:** Patrick D. McLaughlin, Kevin P. Murphy, Lee Crush, Owen J. O'Connor, Joseph P. Coyle, Cressida R. Brennan, Attiya Suhail, Denis Kelly, Michael M. Maher

**Affiliations:** ^1^Department of Radiology, Cork University Hospital, Wilton, Cork, Ireland; ^2^Department of Radiology, University College, Cork, Ireland

## Abstract

*Introduction*. Poor distention decreases the sensitivity and specificity of CTC. The total volume of gas administered will vary according to many factors. We aim to determine the relationship between the volume of retained gas at the time of image acquisition and colonic distention and specifically the presence of collapsed bowel segments at CTC. *Materials and Methods*. All patients who underwent CTC over a 12-month period at a single institution were included in the study. Colonic luminal distention was objectively scored by 2 radiologists using an established 4-point scale. Quantitative analysis of the volume of retained gas at the time of image acquisition was conducted using the threshold 3D region growing function of OsiriX. *Results*. 108 patients were included for volumetric analysis. Mean retained gas volume was 3.3 L. 35% (38/108) of patients had at least one collapsed colonic segment. Significantly lower gas volumes were observed in the patients with collapsed colonic segments when compared with those with fully distended colons 2.6 L versus 3.5 L (*P* = 0.031). Retained volumes were significantly higher for the 78% of patients with ileocecal reflux at 3.4 L versus 2.6 L without ileocecal reflux (*P* = 0.014). *Conclusion*. Estimation of intraluminal gas volume at CTC is feasible using image segmentation and thresholding tools. An average of 3.5 L of retained gas was found in diagnostically adequate CTC studies with significantly lower mean gas volume observed in patients with collapsed colonic segments.

## 1. Introduction

There are a number of fundamental prerequisites for the successful practice of computed tomographic colonography (CTC), namely, satisfactory bowel preparation, faecal and/or fluid tagging, and good luminal distention, as well as thorough interpretation by an experienced radiologist, trained in CT colonography and aided by a modality workstation with specific software packages for CT colonography [1–3]. Inadequate colonic distention, particularly when mucosal surfaces are collapsed and in apposition, may both obscure true mucosal lesions and create false positive pseudolesions thereby decreasing the sensitivity and specificity of CTC [4]. The presence of collapsed segments also contributes to the frequency of repeat CTC examinations and requirement for subsequent optical endoscopic correlation [5].

Methods of insufflation include manual distention with room air [6], manual distention with CO_2_ [5], or a combination of both. Most authors now advocate automated insufflation with CO_2_ [5]. The PROTOCO_2_L device (EZ EM, NY, USA) automatically delivers CO_2_ per rectum and total insufflation volume is limited by an adjustable pressure cutoff switch. Recommended insufflation volumes vary widely in the available literature ranging from 1.5 L to 2 L [3, 7] of gas when delivered manually to a higher median volume of 4.2L in patients who receive automated CO_2_ insufflation with the PROTOCO_2_L device [5].

When performing CTC a large range of insufflation volumes are encountered from patient to patient due to the difference in length and width of an individual's colon, the severity of ileocaecal reflux, and losses due to mucosal resorption and sphincteric incompetence. Burling et al. report a range of administered gas between 2.6 L and 8 L when using the PROTOCO_2_L device [5] and found that the total volume of gas administered was higher in patients with poor distention suggesting loss of gas through anal or ileocaecal incompetence.

Quantitative analysis of final intraluminal gas volumes, estimated from CT images at the time of image acquisition, would allow more accurate definition of the exact volumes of intracolonic gas at the time of CTC and would negate the effect of losses of gas due to mucosal resorption or sphincteric incompetence. There is no record in the literature of studies involving estimation of final intraluminal colonic gas volume from CTC images, and therefore it is unclear if this parameter would predict quality of CTC.

We therefore designed a quantitative study to determine if a relationship exists between the volume of retained gas within the lumen of the colon at the time of image acquisition and image quality of CTC and more specifically with the presence of collapsed bowel segments at CTC.

## 2. Materials and Methods

Our study was approved by the institutional clinical research ethics committee, without requirement for patient consent. A total of 125 consecutive patients who underwent CTC at a single institution between July 2008 and January 2009 were included in the study. Seventeen patients who had metallic hip prostheses (because of severe streak artifact on CT images of pelvis, which precludes estimation of colonic gas volume in the pelvis) were excluded from the study leaving 108 patients for volumetric analysis. The mean age among the study sample was 64 years with a range of 24 to 89 years and male to female ratio was 1 : 1.

Each patient underwent a standardized bowel preparation regimen which consisted of a low-residue diet for 2 days prior to CTC and fluids only from 1 day prior to CTC. A total of 40 mg of Sodium Picosulphate-Magnesium Citrate (Picolax, Ferring Pharmaceuticals Ltd, Berkshire, UK) was administered in four divided doses (10 mg sachets) over a 2-day period. Hyoscine-N-butylbromide (Buscopan, Boehringer, Ingelheim) was not administered routinely as per departmental protocol.

Colonic insufflation was achieved in two steps as per departmental protocol at the time of the study. First, room air insufflation was manually delivered under the care and monitoring of 1 of a group of 13 radiology residents, following rectal placement of a 20 French foley catheter, provided in the PROTOCO_2_L administration set (EZ EM, NY, USA). All residents were trained in colonic insufflation and were experienced in both barium enema and CTC technique. Room air was delivered according to patient tolerance while the patient was placed in a left lateral position. If anal gas leakage was noticed, the foley catheter was exchanged for a larger caliber Miller Air Tip rectal tube (EZ EM, NY, USA). Exchange was necessary in 7 cases (7/108 (6%)). Maintenance insufflation was achieved with CO_2_ using the PROTOCO_2_L device set to maintain a continuous gaseous pressure at the rectum of 25 mmHg. It is worth noting that current departmental protocol now utilises CO_2_ insufflation only. Image acquisition was immediately performed once an adequate and stable rectal pressure was reached.

Patients were imaged pre- and postadministration of 100 mLs of IV iodinated contrast material (300 mg I/mL) in the prone and supine positions, respectively. All studies were performed using a Toshiba Aquilion II 4-slice MDCT scanner (Toshiba Medical Systems Corporation, Otawara, Tochigi, Japan) at 120 kV with a collimation of 3 mm, 2 mm reconstruction interval, rotation time of 0.5 s, and a tube current of 200 mA.

**Figure 1:**
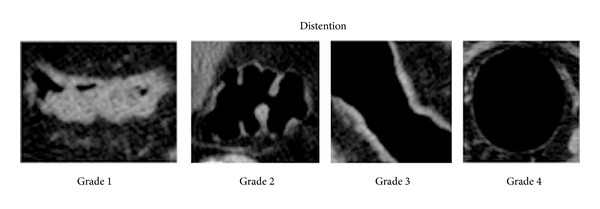
Selected images demonstrating the 4-point colonic distention grading scheme adapted from Burling et al. [5]. Grade 1 indicates complete collapse. Partial collapse (grade 2) was designated when the thickest portion of the haustral folds measured more than 4 mm in width or met within the lumen. Reasonable but suboptimal distention (grade 3) was defined as an easily visible slightly thickened colon wall, and optimal distention (grade 4) was designated when the colonic wall was thin and sharp.

A single gastrointestinal radiologist with 9 years of CTC experience initially interpreted all CTC examinations. Datasets were interpreted using a primary 3D approach with secondary 2D correlation using the Vitrea software suite (Vital Images, Minnetonka, MN, USA). Two radiologists with 7 years and 5 years of experience were blinded to gas volumes and colonic pathologies and scored colonic distention in consensus. We used a semiobjective 4-point scoring system at 6 different locations in the colon, namely, the rectum, sigmoid, descending, transverse, ascending colon, and cecum, as previously described by Burling et al. [5]. Distention grades 1–4 were defined as follows: grade 1, complete collapse; grade 2, partial collapse; grade 3, reasonable but suboptimal distention; and grade 4, optimal distention (Figure 1). The pathological imaging findings were also recorded.

### 2.1. Volumetric Analysis

**Figure 2:**
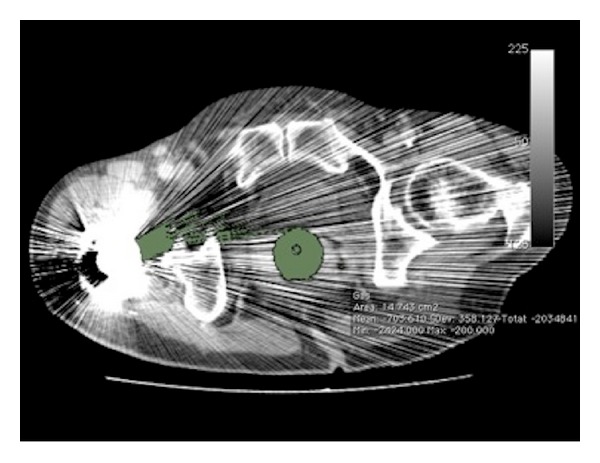
Axial CTC image through the lower rectum demonstrating aliasing artifact from a right hip arthroplasty which spuriously creates pixels with Hounsfield unit values of less than −350 in the soft tissues. This resulted in inaccurate segmentation along the gas column (green ROI). All patients with hip arthroplasty devices were therefore excluded from this study.

Datasets were then imported to OsiriX version 3.3.2 (OsiriX, Geneva, Switzerland), an open source DICOM image analysis suite and PACS workstation designed for the Apple Macintosh platform. All CTC studies were initially reviewed for the presence of imaging artifacts that would create inaccurate segmentation results. Aliasing artifacts from metallic hip arthroplasty devices were a common cause of segmentation error, which typically caused spurious segmentation of the gas column particularly in the rectum (Figure 2).

**Figure 3:**
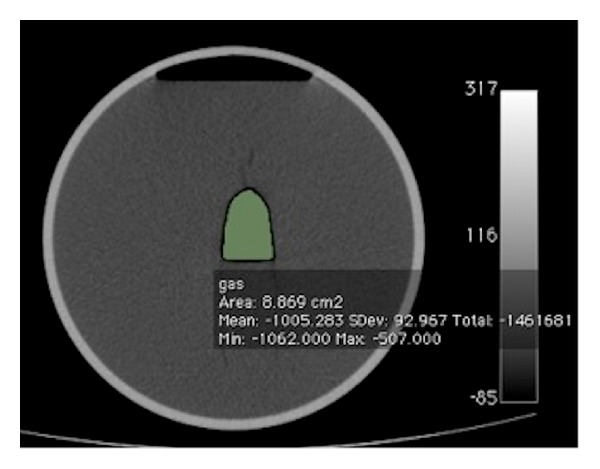
Axial image through a gas containing phantom. Multiple analyses with varying hounsfield values allowed us to find the most accurate range during quantitative estimation to be −350 HU to −1024 HU.

The volume of the column of gas in the colon and small bowel was estimated using the threshold 3D region growing tool available on OsiriX. As with many threshold region growing tools a user defined input of an upper and lower threshold of CT densities was required to commence segmentation. In a phantom study, we previously determined the most accurate range of Hounsfield units for estimating gas volume to be −1024 HU to −350 HU with less than 1% error (Figure 3) [8].

**Figure 4:**
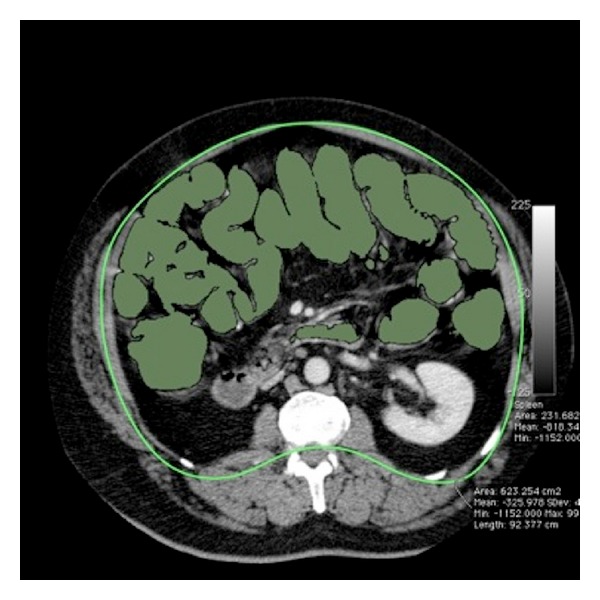
Axial CTC image through the mid-abdomen demonstrating gross ileocecal reflux (green ROI).

Seed points were manually placed in the rectum and region growing using the −1024 HU to −350 HU range was commenced. The segmentation algorithm outputs a 3D region of interest ideally extending from the rectum to the cecum. The presence of ileocecal reflux was then recorded for each patient defined as when the region of interest extended proximally along the small bowel column (Figure 4).

All datasets were reviewed for segmentation errors such as the inclusion of gaseous densities in the stomach and lung or external to the anus and these were corrected manually using the brush tool. All data was collected on an Access 2007 (Microsoft, Redmond, VA, USA) database and statistical analysis was performed using SPSS v16.0 (SPSS Inc, Chicago, USA).

## 3. Results

The mean intraluminal gas volume contained within the colon and small bowel was 3.3 L with range of 1.1 to 6.8 L. Mean gas volume was slightly higher in males (3.4 L) when compared with females (3.2 L), but this difference did not reach statistical significance.

Ileocecal reflux was found in 78% of patients (*n* = 84). Patients with ileocecal valve reflux were found to have significantly higher gas volumes when compared with patients who had competent ileocecal valves (3.4 L versus 2.6 L, *P* = 0.014). No significant difference in distention scores was found between patients with ileocecal competence and incompetence.

Diverticulosis was found in 40% of patients. Nondistended segments were more frequently found in this group, but there was no significant difference in average distention scores or intracolonic gas volume when compared with the group of patients unaffected by colonic diverticulosis.

A total of 38 patients (35%) had one or more nondistended colonic segments (Table 1). The sigmoid colon had the lowest mean colonic distention score and was the most frequently nondistended segment representing 44% of all collapsed segments. Total gas volumes were significantly lower in patients with one or more nondistended colonic segments when compared with patients with fully distended colons (2.6 L versus 3.5 L, *P* = 0.031).

## 4. Discussion

Gaseous distention of the colon is required to prevent colonic mucosal surfaces from collapsing together and opposing each other which would potentially obscure true lesions or create pseudolesions [1]. Colonic distention directly impacts on the sensitivity and specificity of CTC as a diagnostic test and therefore is a critical determinant of diagnostic quality at CTC [1–4, 9]. In general, the colon has a high compliance when gas is administered under low pressure; in this situation most of the gas insufflated into the colon contributes to the expansion of the colon with little increase in pressure. With sustained insufflation, as colonic volume increases, so does intraluminal gas pressure, and Laplace's law predicts that colonic wall tension will also significantly increase leading to an elevated risk of perforation [10].

Colonic luminal perforation is perhaps the most serious adverse event associated with CTC. The incidence of symptomatic colonic perforation associated with CTC has previously been found to be 0.05% in a series of 17,067 patients [11] and 0.06% in a series of 11,870 patients [12] and 3458 patients, respectively [13]. Increased intraluminal pressures can lead to colonic rupture. An adult human cadaveric cecum exposed to less than 40 mmHg of intraluminal pressure generally does not rupture; however a cecum exposed to more than 150 mmHg of pressure always ruptures [14]. Tzelepis et al. previously estimated that the upper limit of safe intraluminal colonic pressure is 80 mmHg, as perforation can occur at pressures greater than 140 mmHg [15].

In day-to-day practice, to decrease the risks of perforation, colonic gas volumes and intraluminal pressures should be maintained at the lowest acceptable levels that yield diagnostically adequate distention. We reviewed the available literature in an attempt to determine an acceptable range of colonic insufflation volumes and found that all papers, which correlate distention with colonic gas volumes, quote the total insufflated volume. This is the volume of gas administered per rectum and includes intracolonic gas, gas refluxed into the small bowel, and gas lost through anal incompetence and mucosal reabsorption.

As previously stated we found a large range from 2.3 L to 8 L [5, 14] of total automated CO_2_ insufflation volumes quoted in the literature likely relating to unaccounted gas losses due to anal/ileocecal incompetence or mucosal absorption. It is clear that large unaccounted losses of gas would significantly limit any meaningful correlation between luminal distention and total insufflated volume; therefore our study primarily aims to negate the effect of lost gas by employing image segmentation software to quantify only the volume of retained intraluminal gas. Another advantage of this means of quantifying intraluminal colonic and small bowel gas volume is that this measure represents intraluminal gas volume at the time of image acquisition. It can be reliably measured by image segmentation software at most workstations, without any change to CTC technique or method of gas insufflations. It is our belief that this objective measure is potentially very important, in the evaluation of any modification of CTC technique being developed to improve quality of CTC.

In our population the mean gas volume contained within the colon and small bowel was 3.3 L. There was a large range of intraluminal gas volumes from 1.1 to 6.8 L. Patients with ileocecal incompetence (78%) were found to have significantly higher gas volumes than patients who had competent ileocecal valves. We insufflated gas manually according to patient tolerance and maintained a constant colonic pressure of 25 mmHg during the procedure suggesting that patients with ileocecal reflux tolerated increased volumes of gas or had lower periprocedural intraluminal colonic pressure resulting in an increased insufflation volume. Despite larger intraluminal gas volumes no significant difference in distention score was found between patients with ileocecal competence and incompetence.

We found that total gas volumes were significantly lower in patients who had one or more nondistended segments. A mean intraluminal gas volume of 2.6 L was seen in patients with one or more nondistended segments compared with 3.5 L in those patients with fully distended, diagnostically adequate colons. Diverticulosis was found in 38% of patients, and there was no significant difference in average distention scores when compared with the group of patients without diverticulosis.

There are obvious limitations when applying our study results in everyday practice. Firstly we have limited our analysis to intraluminal gas volumes only; therefore we cannot provide an accurate guideline for the total insufflation volume to be administered at CT. In addition, the total insufflation volume may vary with different insufflator types. Actual insufflation volumes should be individually increased in cases of anal and ileocaecal incompetence. We also cannot account for the volume of gas that was contained within the colon prior to insufflation. However, the methodology of calculating intraluminal gas volume described in this study may be of value as a reproducible index of quality. This measurement can be made after CTC without any modification to CTC technique; any risk of adverse outcome for the patient or any prolongation of the procedure and intraluminal gas volume could represent an additional measurable endpoint and possibly an indicator of quality of CTC examination. Finally, correlation between accuracy of abnormal lesion detection on CTC and adequacy of gaseous distention through volumetric measurements is an avenue of future research that should be considered.

## 5. Conclusion

In this study, analysis of retained intraluminal gas volumes at CTC demonstrates that higher intraluminal gas volumes are associated with a decreased incidence of colonic nondistention. The mean colonic intraluminal gas volume in patients with diagnostically adequate colonic distention was 3.5 L. This volume was significantly higher than the mean volume in patients with one or more nondistended or collapsed segments of colon (2.6 L). Intraluminal gas volumes are higher by an average of 800 ccs in patients with ileocecal valve incompetence. Finally the authors conclude that colonic insufflation is a dynamic process [14] and at all times clinical adjustments should be made in real time for anal incompetence and ileocecal valve reflux, and total insufflation volumes should be moderated according to patient tolerance.

## Figures and Tables

**Table 1:** The average distention score for each of the 6 colonic segments examined. The total number of collapsed (grade 1) segments found is also reported.

Segment of colon	Rectum	Sigmoid	Descending colon	Transverse colon	Ascending colon	Cecum
Average distention	2.35	1.86	2.6	2.9	2.93	2.95
No. of collapsed segments (grade 1)	20	22	8	0	0	0
